# Stochastic fracture of additively manufactured porous composites

**DOI:** 10.1038/s41598-018-33863-4

**Published:** 2018-10-18

**Authors:** Özgür Keleş, Eric H. Anderson, Jimmy Huynh, Jeff Gelb, Jouni Freund, Alp Karakoç

**Affiliations:** 1 Chemical and Materials Engineering Department, San Jose State University, San Jose, CA 95192 USA; 2 Sigray, Inc., Concord, CA USA; 3 Department of Mechanical Engineering, Aalto University, Espoo, Finland; 4 Civil and Environmental Engineering, University of California, Los Angeles, USA

## Abstract

Extrusion-based fused deposition modeling (FDM) introduces inter-bead pores into dense materials, which results in part-to-part mechanical property variations, i.e., low mechanical reliability. In addition, the internal structure of FDMed materials can be made porous intentionally to tailor mechanical properties, introduce functionality, reduce material consumption, or decrease production time. Despite these potential benefits, the effects of porosity on the mechanical reliability of FDMed composites are still unclear. Accordingly, we investigated the stochastic fracture of 241 FDMed short-carbon-fiber-reinforced-ABS with porosity ranging from 13 to 53 vol.% under tensile load. Weibull analysis was performed to quantify the variations in mechanical properties. We observed an increase in Weibull modulus of fracture/tensile strength for porosity higher than ~40 vol.% and a decrease in Weibull modulus of fracture strain for an increase in porosity from 25 to 53 vol.%. Micromechanics-based 2D simulations indicated that the mechanical reliability of FDMed composites depends on variations in bead strength and elastic modulus of beads. The change in raster orientation from 45°/−45° to 0° more than doubled the Weibull modulus. We identified five different types of pores via high-resolution X-ray computed tomography. A 22% and 48% decrease in carbon fiber length due to extrusion was revealed for two different regions of the filament.

## Introduction

Additive manufacturing (AM) is increasingly used for applications that require metal, polymer, ceramic, and composite materials^1,2^. The unprecedented design flexibility of AM allows for mass customization, distributed manufacturing, improved automation, and on-demand production, while decreasing material consumption^3–6^. As a result, AM has seen growth in many industries including aerospace, defense, automotive, medical, dental, construction, consumer-grade manufacturing, fashion, and open source designs^1–4,7–9^. The large material space and design space of additively manufactured (AMed) products resulted in an annual growth rate of approximately ~26% over the last three decades^10^. Among various AM techniques, fused deposition modeling (FDM) is the most common technique due to its simple extrusion-based design and availability of cost-effective materials. However, fused deposition modeled (FDMed) polymers and composites contain processing defects such as inter-bead and inner-bead pores that decrease mechanical performance^11–14^. These pores have a range of sizes that cause variations in mechanical properties, which thus decrease mechanical reliability^12^. Despite the increasing use of the FDMed composites, the mechanical reliability of FDMed dense short-carbon-fiber-reinforced (SCFR) composites, and the effects of porosity on the stochastic fracture of FDMed SCFR composites are still unclear. In order to ensure the reliable and safe use of FDMed SCFR composites, we require a better understanding of the variations in their mechanical properties.

FDM systems–also known as fused filament fabrication–use cost effective polymers and polymer composites, which accounted for the sale of more than million FDM systems between 2013 and 2017^10,15^. Reports indicate that AM of polymers/composites is financially competitive for productions up to 10,000 parts compared to the traditional injection molding process^7,16^. In addition, FDM has the potential to disrupt mass production due to its ability to produce structures that are porous, topologically optimized, or multi-material without any tooling. Consequently, AM enables a manufacturing trend towards mass customization, allowing products to be tailored and optimized for individual needs. For example, the porosity and stiffness of a shoe sole can be adjusted based on the weight of a person; hard tissue scaffolds can be optimized for strength and bone growth based on a patient’s age and sex. Intentional porosity also reduces material usage, part weight, and build time. However, these modifications in the internal structure of AMed products result in mechanical reliability problems. Therefore, interrelationships between AM processing parameters, structure, and mechanical reliability must be known in order for AM to compete/replace mass production and enable safer use of customized products.

Various polymer composites with reinforcing phases have been produced using FDM including, short fibers^14,17–19^, continuous fibers^20,21^, graphite^22^, and graphene^23^. Studies have reported the effects of FDM process parameters, carbon fiber content, fiber length, and raster orientation on the mechanical properties of FDMed SCFR-acrylonitrile butadiene styrene (ABS)^14,17–19,22,24^. These studies, however, were performed on sample sizes (N) ranging from 5 to 12, which are not enough to investigate stochastic fracture of FDMed SCFR-ABS. Large number of specimens (N > 20) are required to obtain statistically significant fracture data that quantify the variations in mechanical properties with a low relative error^25^. The scatter in Weibull modulus rapidly decreases with increasing N up to 20 and the reduction in scatter becomes gradual for N > 40. For instance, the relative error decreases by ~4% at 90% confidence level for an increase in N from 27 to 40, which represents the range of sample sizes in this study. Whereas, the relative error decreases ~5% for an increase in N from 40 to 80^25^.

The variations in mechanical properties can be quantified by Weibull analysis, which is commonly used for ceramic materials^26–28^. Weibull distribution is a flexible function that can be fitted to any kind of data. Specifically, two-parameter Weibull distribution is widely used to quantify mechanical reliability^26,27^:

1 $$F(\sigma ,V)=1-\exp [-\,V/{V}_{0}{(\sigma /{\sigma }_{0})}^{m}]$$

where the probability of failure (*F*) is related to the volume of the specimen (*V*) and applied stress (*σ*). The Weibull modulus (*m*) is the shape parameter related to the variation in mechanical property data. Higher *m* means lower variation in data and lower *m* means higher variation. For example, traditional ceramics have *m* = 3–5; engineering ceramics have *m* = 10–25; metals have m = 90–100^28,29^. The characteristic strength (*σ*_0_) corresponds to the failure probability of 63%. A normalizing volume (*V*_0_) equal to the volume of the specimen (*V*) can be assumed for the same size specimens. As a result, Weibull distribution relates the applied stress to the probability of failure; thus, it provides a practical design criterion to ensure specific reliability levels for products.

Weibull analysis has been used for AMed ceramics to quantify the high variability of these materials’ fracture strength^30–33^. We also used Weibull statistics to quantify the stochastic fracture of FDMed ABS, which showed that the variation in fracture stress of FDMed ABS can be as high as for technical ceramics, *m* = 26^11^. In addition, mechanical reliability depended on the build orientation, change in local structure (addition of a hole), and test specimen shape^11^. For example, we reported a Weibull modulus of 69 for the XZ build orientation and 26 for the C + 45 orientation^11^. Moreover, we showed that a decrease in inter-bead porosity increased the Weibull modulus for SCFR-ABS from 25 to 57^12^.

We investigated the effects of porosity and raster orientation on the stochastic fracture of FDMed SCFR-ABS. Five different porosity levels between 13 and 53 vol.% were tested. Seven batches of tensile tests were performed on at least 27 specimens per batch, a total of 241 tests. Additionally, we tensile tested the as-received filaments and extruded filaments, a total of 90 tests. The SCFR-ABS composites produced for this study were brittle or had limited plasticity. Therefore, we applied Weibull statistics to quantify the variations in mechanical properties of the composites. The experimental strength data were used to set up a micro-mechanical simulation framework, which revealed the origin of the variations in mechanical properties. We performed fractography using scanning electron microscopy (SEM) and used micro X-ray microscopy to observe the various types of pores in the FDMed composites.

## Results and Discussion

### Porosity in SCFR-ABS

**Figure 1:**
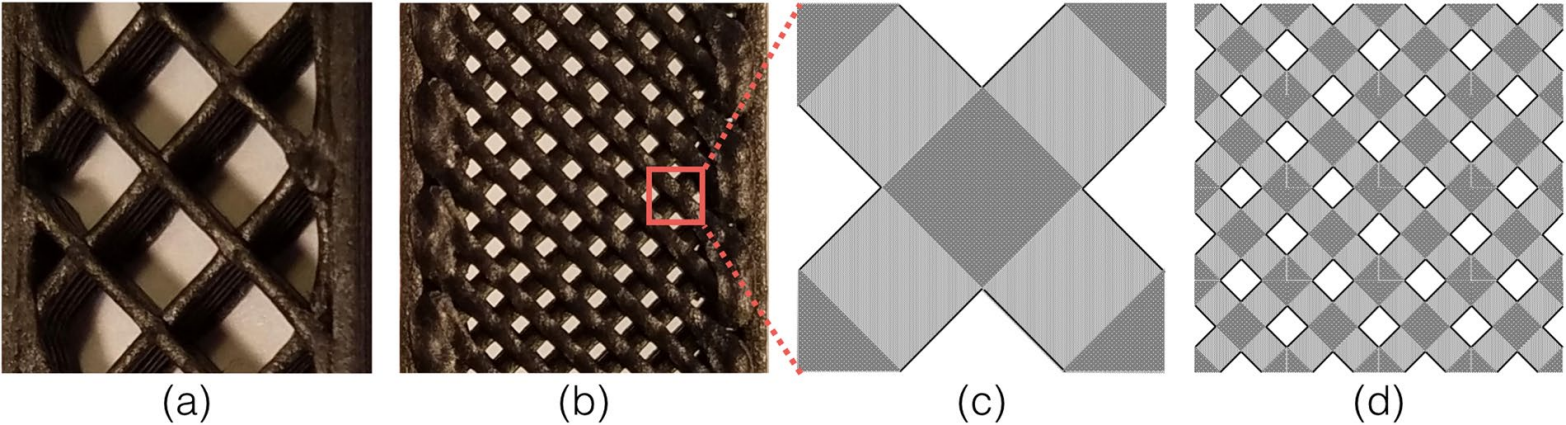
Internal structures of the composites showing infill% of (**a**) 20% and (**b**) 80%; (**c**) a representative volume element composed of rectilinear beads, and (**d**) simulated 80% infill structure representing the experimental infill structure in (**b**).

**Figure 2:**
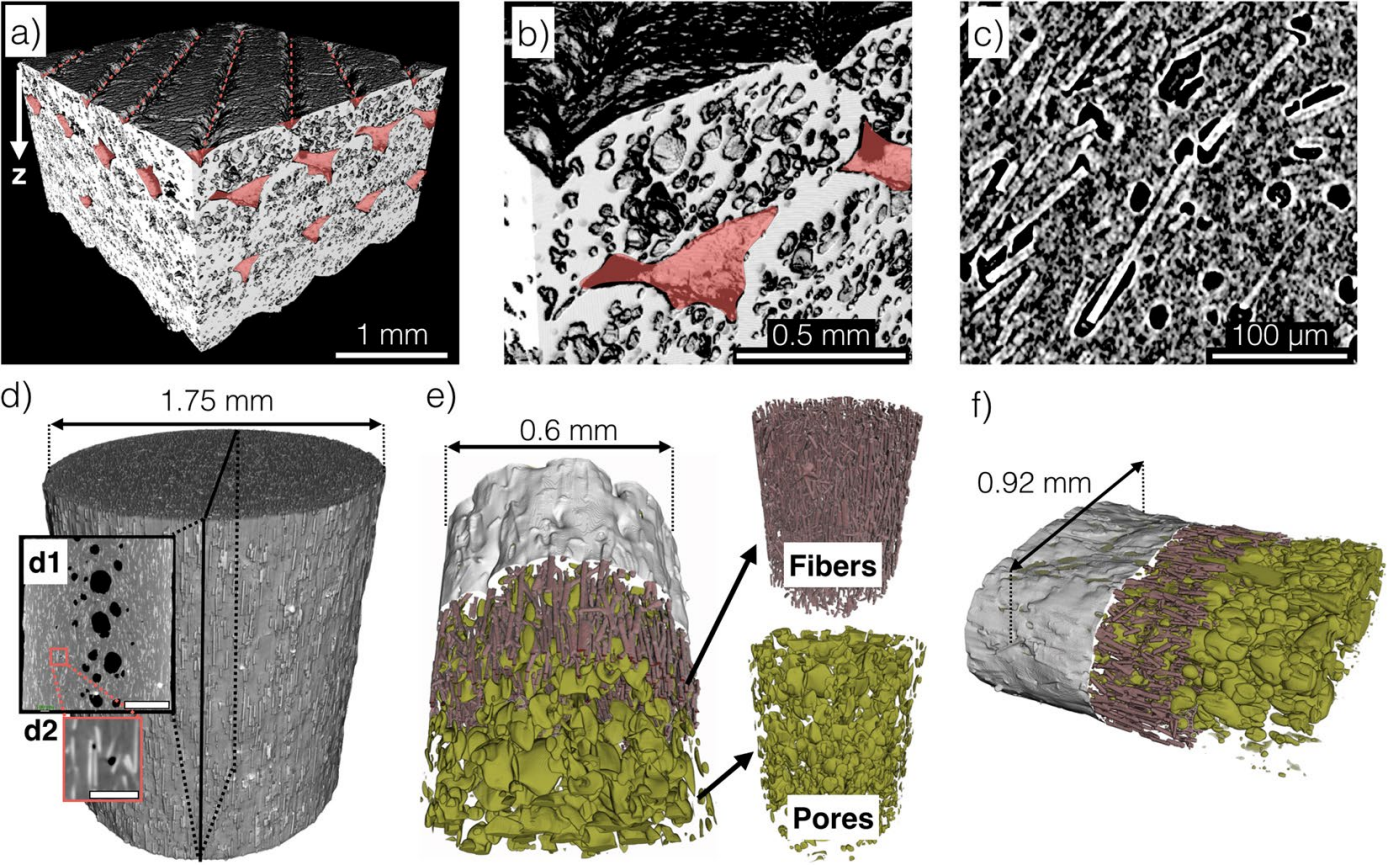
X-ray computed tomography images of (**a**) infill = 100% composite showing inter-bead porosity as red shaded areas, (**b**) beads with inter- and inner-bead porosity, (**c**) carbon fibers and pores at the fiber ends and around fibers, (**d**) as-received filament, (**e**) extruded filament with fiber and pore distribution, and (**f**) extruded filament that was deposited on the hot bed. ABS is shown in shades of gray in (**a**) to (**f**). Black represents pores in (**c**) and (**d**). Dark green represents pores and dark red represents carbon fibers in (**e**) and (**f**). The porosities (vol.%) are (**d**) 5, (**e**) 20, and (**f**) 22. The carbon fiber content (vol.%) are (**d**) 7, (**e**) 6, and (**f**) 10. Scale bars represent 500 *μ*m for d1 and 100 *μ*m for d2. Isotropic voxels of linear dimensions are 4.4 *μ*m for (**a**) and (**b**), 0.9 *μ*m for (**c**), 1.0 *μ*m for (**d**) and (**f**), and 0.83 *μ*m for (**e**).

We identified five different types of pores in FDMed SCFR-ABS composites using digital images and X-ray computed tomography (Figs 1 and 2). These pores were: (a) intentional porosity for infill <100%, (b) inter-bead porosity due to the lack of complete geometric filling between beads, (c) inner-bead porosity inherited from the as-received filament, (d) inner-bead porosity due to flow incompatibility between ABS and carbon fiber, and (e) inner-bead porosity due to pores generated during high-temperature extrusion. The intentional porosity in our systems was generated by setting the infill percentage in the slicer program. The infill% defines the percentage of the volume filled by the extruded material. Therefore, an infill% = 100 defines a nominally dense material and an infill% < 100 generates rectilinear lattice structures. Example structures for infill = 20% and 80% are given in Fig. 1a,b. These intentional pores are seen as square pores that are the largest in our FDMed composites.

Nominally dense FDMed materials contain inter-bead pores because filament was extruded through a circular nozzle and the resulting deposited beads cannot completely fill the volume of the CAD design. See red-shaded areas in Fig. 2a,b. The inter-bead porosity can be reduced by deposition path algorithms^34^, by using thermally expandable second-phases in filament^35^, and by over-extruding to fill inter-bead pores at an infill% higher than 100%. We did not use any approach to reduce inter-bead porosity. Therefore, inter-bead pores are the second largest defects in our FDMed composites. However, these pores can be as long as the deposited beads since they are adjacent to the beads. The inter-bead pore thickness and width mainly depend on the nozzle size, layer thickness, and extrusion temperature. The total porosity of our dense specimens was 15 vol.% for material 1 (M1) and 13 vol.% for material 2 (M2); M1 and M2 were the same SCFR-ABS material purchased one year apart. If we exclude the inherited ~5 vol.% porosity from the filament, the maximum inter-bead porosity was between 8 to 10 vol.%.

The pores that are inside the beads are called inner-bead pores^14^. We observed large (Fig. 2b) and small (Fig. 2c) inner-bead porosity. The large inner-bead pores (>50 *μ*m) were inherited from the as-received filament because pores were present along the central axis of the filament (Fig. 2d1). The 3-D X-ray CT reconstruction showed that the filament contained ~5 vol.% porosity (*P*). These pores were entrapped during the deposition and printed along with the carbon fibers. The sphere-like pores in the as-received filament were elongated during the extrusion (Fig. 2d,e). Smaller pores at the fiber-ends were also present in the filament before (Fig. 2d2) and after the extrusion (Fig. 2c). These smaller pores at the fiber-ends were suggested to be generated during the extrusion due to the flow incompatibility of carbon fibers and ABS^14^. An increase in nozzle temperature from 200 °C to 240 °C has been previously reported to increase the inner-bead porosity in SCFR-ABS^22^. We used a nozzle temperature of 235 °C, which could have caused inner-bead porosity in our study. The relative ratios of these inner-bead pores are challenging to quantify.

The extrusion of the as-received filament increased its porosity from 5 vol.% to ~20 vol.% for air-extruded filament (Fig. 2e) and to ~22 vol.% for deposition on the hot print bed (Fig. 2e). These porosity values were obtained from the X-ray CT data. The porosities of extruded/deposited filament are higher than the total porosity 13–15 vol.%. The difference between the total *P* and the extruded/deposited filament is due to (a) the decreasing porosity in the Z-direction, i.e., inner-bead porosity decreased as the extrusion head deposited on the layers away from the hot bed (Fig. 2a); (b) fluctuating porosity along the as-received filament, i.e., there were large-pore-free regions and high-porosity regions along the as-received filament (see Fig. 2d1). We performed 15 density measurements at a section of ~1 m for M1 filament and observed a density of 1.23 ± 0.06 g/cm^3^; one of these densities was 1.07 g/cm^3^ compared to the range of 14 specimens −1.20 g/cm^3^ to 1.27 g/cm^3^. Accordingly, the increase in porosity after extrusion can also be caused by the porosity variations at different regions of the filament in addition to the local porosity fluctuations and heat induced pores. In the rest of the discussion, porosity refers to total porosity.

### Strength and structure relationship in SCFR-ABS

**Figure 3:**
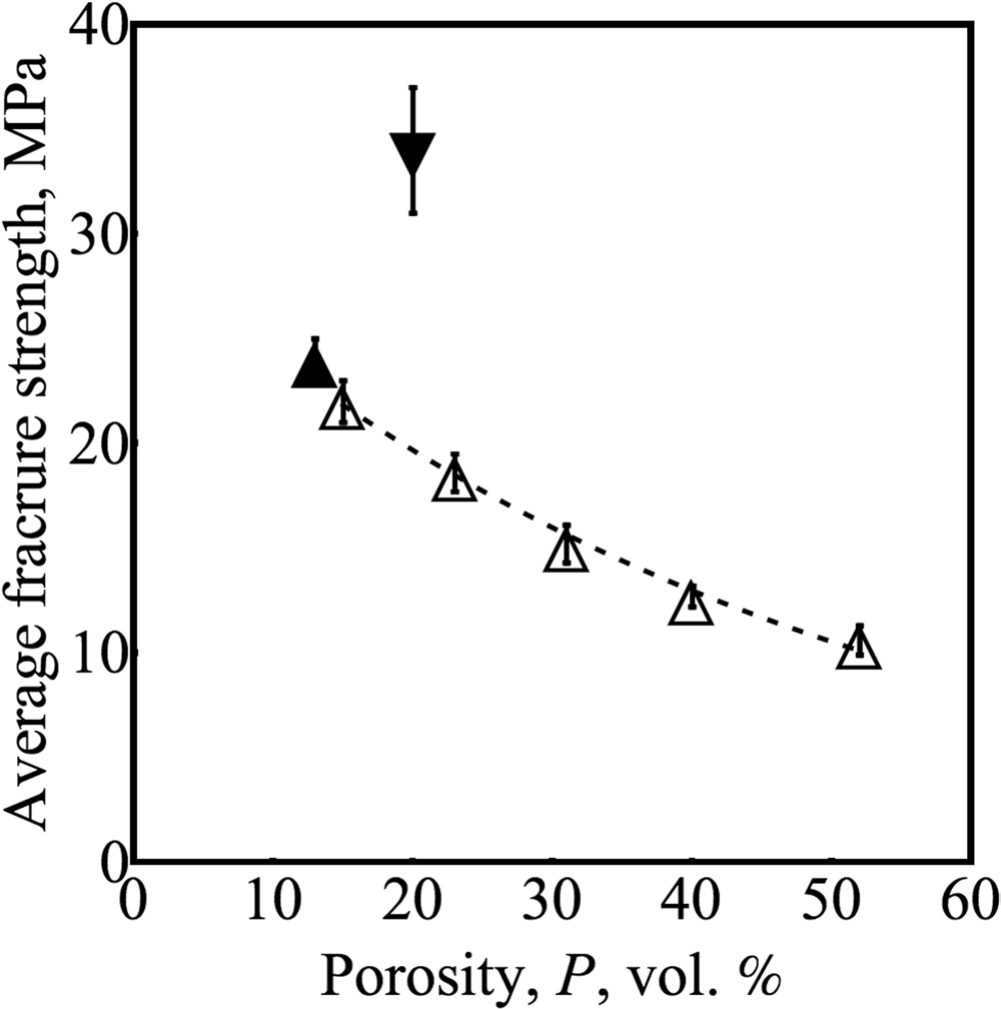
Effect of porosity on the average fracture strength of FDMed SCFR-ABS composites, △ represents the M1, ▲ represents M2 with 100% infill, and ▼ represents M2 at 100% infill in 0° raster orientation. Dotted line represents the fit to *σ*_0_ Exp[−bP] (*σ*_0_ = 30 MPa, *b* = 0.02) with an R^2^ = 0.99. Error bars represent one standard deviation.

**Figure 4:**
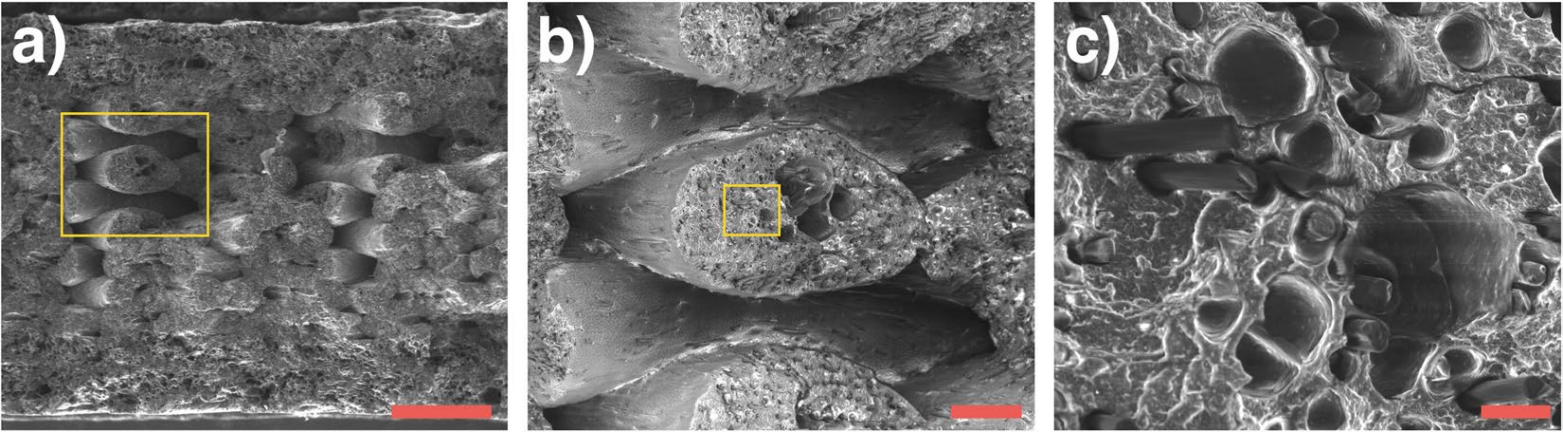
SEM micrographs of the fracture surface of FDMed SCFR-ABS with a 100% infill including (**a**) inter-bead porosity, (**b**) inherited porosity, and (**c**) inner-bead porosity. Micrograph (**b**) shows the magnified yellow rectangle in (**a**) and (**c**) shows the magnified yellow rectangle in (**b**). Scale bars represent (**a**) 1 mm, (**b**) 200 *μ*m, and (**c**) 20 *μ*m.

We performed tensile tests on seven different sets of SCFR-ABS: each set contained at least 27 specimens, a total of 241 tests (Table 1). The average fracture strength (<*σ*_*f*_>) of the nominally dense specimens was ~23 MPa. Increasing porosity (decreasing infill%) decreased the <*σ*_*f*_> and tensile strength (TS) (Fig. 3). The effects on infill% on TS of FDMed ABS were previously investigated by Fernandez-Vicente *et al*.^36^. The TS of ABS was found to be 16, 20, 36 MPa for 20, 50 and 100% rectilinear infills, respectively. These TS data for ABS were represented with the function ${\sigma }_{P}\simeq 15+0.002{x}^{2}$, where *σ*_*P*_ is the TS and *x* is the infill density. We fit the same function to the TS of SCFR-ABS and obtained ${\sigma }_{P}\simeq 11+0.001{x}^{2}$ with an R^2^ = 0.99. Compared to the ABS, TS of the SCFR-ABS showed less dependence on the infill%, which is due to the inner-bead pores (Fig. 4a) and inter-bead pores (Fig. 4b,c). The inner-bead porosity concentrates applied stress inside the beads. Accordingly, TS of our composites depended on both inter-bead and inner-bead porosity, which, in turn, decreased the TS dependence on the infill%.

**Table 1:** Summary of mechanical properties for material 1 and 2 (M) with sample size N that are build in raster orientation (R, 45 = −45/45°, 0 = 0°) with an infill (I, %) and porosity (P, vol.%). E is the elastic modulus in GPa.

M	N	R	I	P	E	*σ*_*f*_		TS		*ε*_*f*_		*ε*_*TS*_	
						m	*σ*_0_	m	*σ*_0_	m	*σ*_0_	m	*σ*_0_
1	27	45	100	15 ± 4	2.5 ± 12	24 (19–30)	23	23 (18–29)	23	11 (9–14)	3.7	28 (22–36)	3.0
1	37	45	80	25 ± 3	2.1 ± 11	25 (20–30)	20	24 (20–30)	20	21 (17–26)	3.2	26 (21–32)	2.9
1	39	45	60	34 ± 2	1.9 ± 09	18 (15–21)	16	18 (15–21)	16	15 (12–19)	2.4	18 (15–22)	2.3
1	38	45	40	41 ± 1	1.7 ± 08	36 (30–44)	13	41 (34–49)	13	11 (9–14)	2.3	12 (10–15)	2.3
1	40	45	20	53 ± 1	1.5 ± 08	39 (32–48)	11	41 (33–51)	11	8 (7–10)	1.9	9 (7–11)	1.8
2	30	0	100	23 ± 3	5.0 ± 4	40^*^ (30–52)	32^*^	46^*^ (36–61)	33^*^	17 (13–22)	2.4	19 (14–25)	2.0
2	30	45	100	13 ± 3	3.0 ± 3	19 (15–24)	24	19 (15–25)	24	9 (8–12)	2.8	8 (6–10)	2.7

Weibull modulus (m) and scale parameter (*σ*_0_) of the fits to fracture stress (*σ*_*f*_), tensile strength (TS), nominal strain at break (*ε*_*f*_), and strain at tensile strength (*ε*_*TS*_). The 90% confidence intervals are given in parenthesis.

^*^Results represent the estimates from censored data excluding the top 10 highest strength values.

We chose to present our data based on porosity instead of infill%, because *P* is a physical property; whereas, infill% describes a processing parameter that is not directly related to the porosity. Our composites showed brittle behavior with no to limited plasticity. This brittle behavior can be attributed to the inner-bead porosity that introduces strong pore-to-pore stress interactions and also the pores at the fiber-ends that reduce the toughening due to fibers. The sets with the lowest three porosity levels (P = 52, 40, and 31 vol.%) showed brittle behavior, while the differences between the strain at tensile strength (*ε*_*TS*_) and nominal strain at break (*ε*_*f*_) were statistically the same (Table 1). The rest of the specimens showed limited plasticity with *ε*_*f*_ less than 4.5%. Therefore, we used the exponential function^37,38^ −*σ*_*f*_ = *σ*_0_Exp[−*bP*] –to describe the relationship between P and *σ*_*f*_ (Fig. 3). This exponential function is widely used to represent relationships between *P* and *σ*_*f*_ in brittle materials, where the parameter *b* represents the sensitivity of *σ*_*f*_ to *P*. Other studies have reported the *b* value as between 0.015 and 0.074 for various porous materials including glass, alumina, zirconia, and compacted cellulose^39^. Our exponential fit to the SCFR-ABS fracture data had a *b* value of 0.02, which is between the values for porous glass and alumina^39^.

Raster orientation defines the spatial distribution of inter-bead porosity and fiber orientations that strongly affect mechanical properties in FDMed materials^11,22^. In this context, we investigated the effects of change in raster orientation from 45°/−45° to 0° on the strength of composites. The beads were parallel to the applied tensile load in the 0° raster orientation. Accordingly, load transfer to carbon fibers was more effective because the extrusion process aligns the fibers along the deposition path (Fig. 2e,f). In 0° orientation, inter-bead porosity is also parallel to the applied load; thus, the stress concentration due to inter-bead porosity is lower compared to 45°/−45° raster. As a result, the change in raster orientation from the 45°/−45° to 0° increased the TS by 42% and increased the elastic modulus by 67% (Table 1).

**Figure 5:**
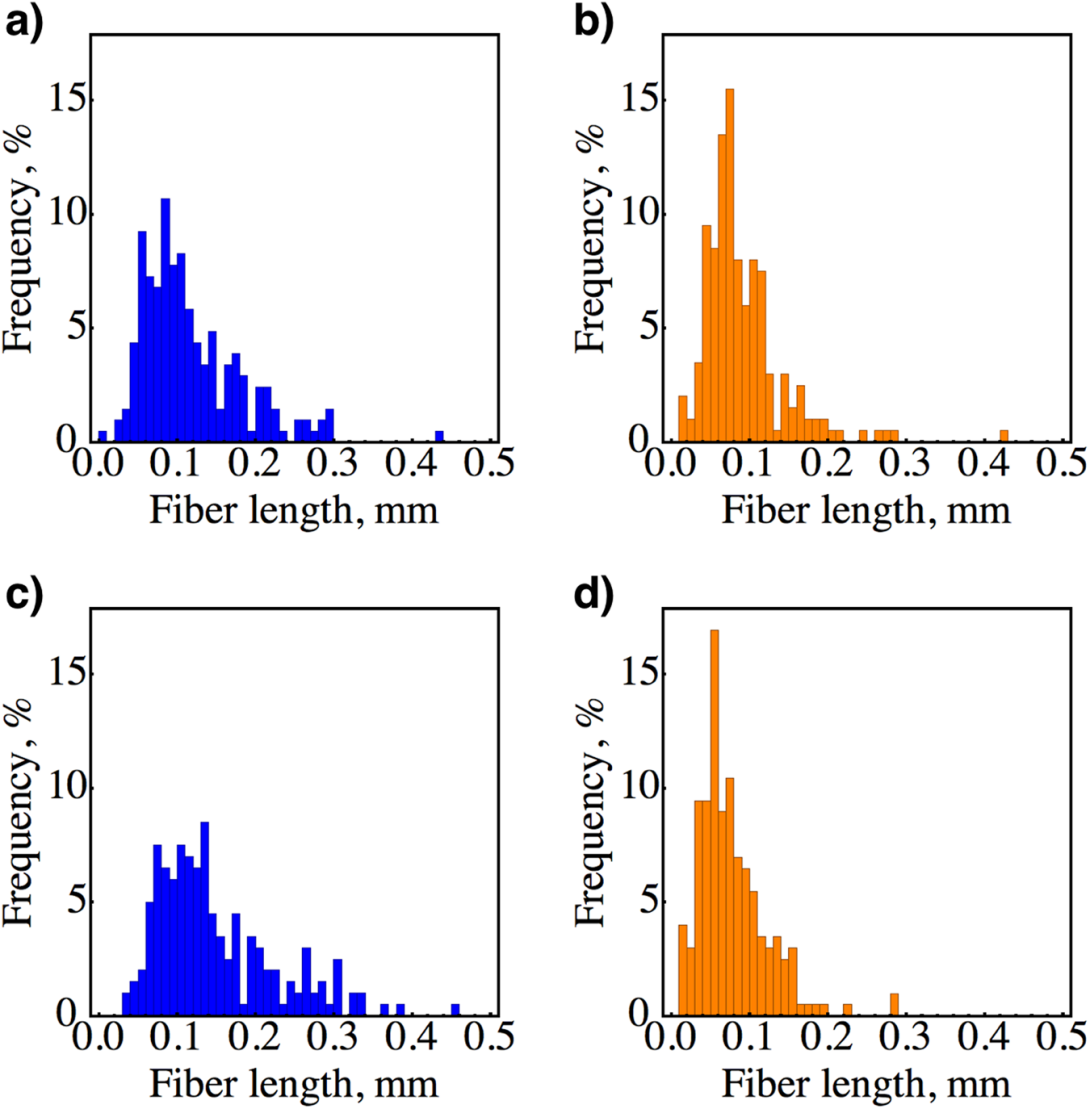
Fiber length distributions for raw filament (**a**) and (**c**), and extruded filament (**b**) and (**d**). The mean carbon fiber lengths and standard deviations are (**a**) 117 ± 65 *μ*m, (**b**) 91 ± 52 *μ*m, (**c**) 148 ± 76 *μ*m, and (**d**) 77 ± 44 *μ*m. The data in (**b**) was generated from the same same region as (**a**) and the data in (**d**) was generated from the same region as (**c**). In other words, (**a**–**d**) represent the same region along the filament within ~3 cm.

However, in this study, the TS of 0° raster composites is ~30% lower than the results of Tekinalp *et al*.^14^ in the same raster orientation, which is mainly due to our shorter average fiber length of 91 ± 52 *μ*m or 77 ± 44 *μ*m compared to ~250 *μ*m and 23 vol.% porosity (Table 1). Extrusion through the 0.6 mm nozzle decreased the as-received average fiber length from 117 ± 65 *μ*m to 91 ± 52 *μ*m (a 22% decrease) at one location of the M1 filament and at another location the fiber length decreased from 148 ± 76 *μ*m to 77 ± 44 *μ*m (a 48% decrease). The change in fiber length distributions is shown in Fig. 5. The average fiber diameter was ~6–8 *μ*m based on the X-ray CT analysis. From a critical fiber length (*l*_*c*_) point of view, the minimum fiber length should have been between 216 *μ*m and 576 *μ*m according to *l*_*c*_ = *σ*_*fiber*_*d*/2 *τ*_*m*_^40^, since we assumed the carbon fiber strength *σ*_*fiber*_ = 2.16 GPa^41^ and fiber-matrix interface shear strength *τ*_*m*_ = 30–15 MPa (≤tensile strength of ABS^14^). Effective strengthening of ABS with short fibers requires fiber lengths higher than *l*_*c*_, i.e., higher in aspect ratio^42^. For example, a fiber length to *l*_*c*_ ratio of 10 would strengthen the ABS up to ~95% of continuous fiber strengthening^43^.

### Effect of structure on the Weibull modulus of SCFR-ABS

**Figure 6:**
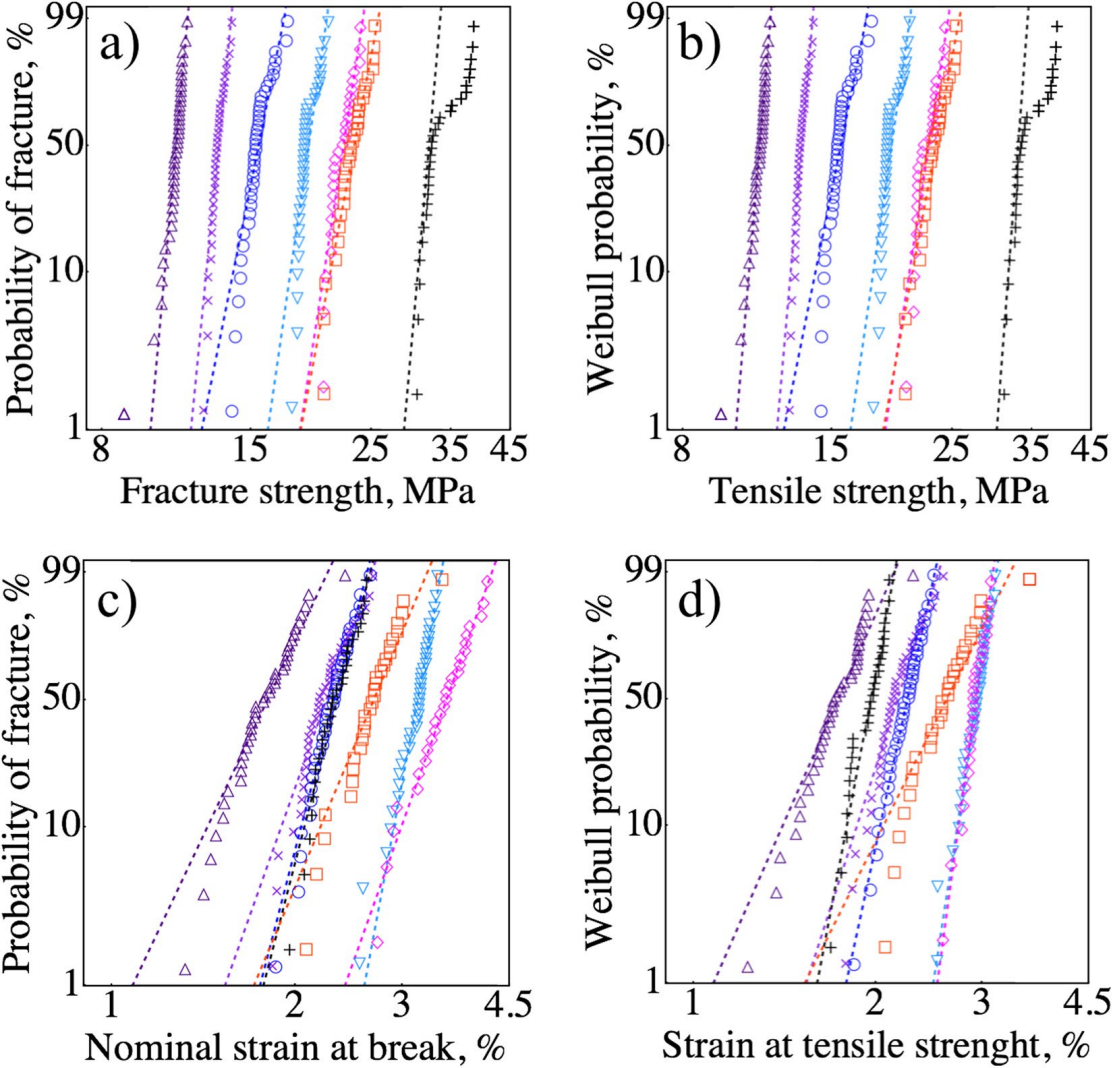
Weibull plot showing the variations in (**a**) fracture strength, (**b**) tensile strength, (**c**) nominal strain at break, and (**d**) strain at tensile strength of SCFR ABS for M1 P = 53 vol.% Δ, P = 41 vol.% ×, P = 34 vol.% ◯, P = 25 vol.% ∇, P = 15 vol.% ◇, for M2 P = 13 vol.% □, and P = 23 vol.% + .

**Figure 7:**
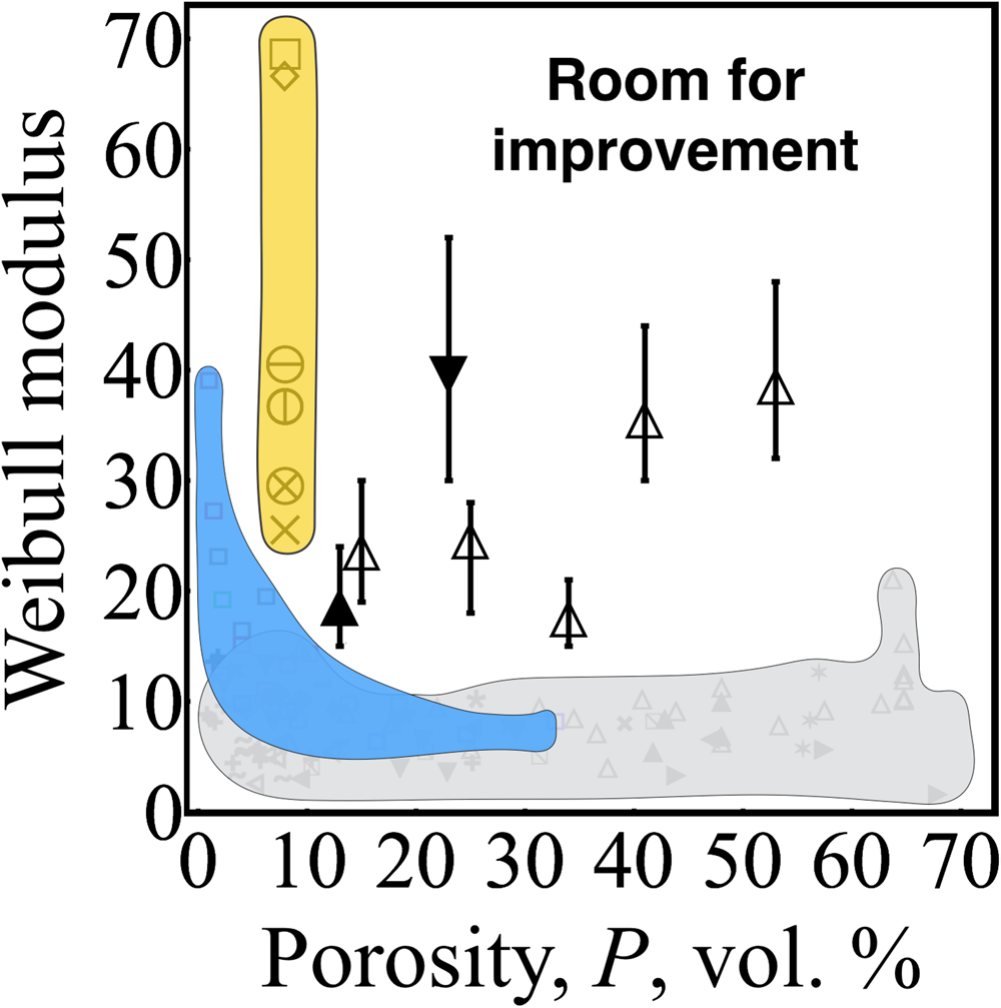
Effect of porosity on the Weibull modulus of FDMed-SCFR composites; Δ is for M1, ▲ is for material 2 at 100% infill, and ▼ is for M2 at 100% infill in 0° raster orientation. Error bars represent 90% confidence intervals. Yellow represents moduli of FDMed ABS built in different orientations^11^. Gray represents experimental moduli of various porous ceramics^52^. Blue represents simulated modulus of brittle porous materials^52^.

We performed Weibull analysis to quantify the variations in *σ*_*f*_, TS, *ε*_*f*_, and *ε*_*TS*_. These variations were visualized in Weibull plots, with the fitted Weibull distributions shown as dotted lines (Fig. 6). The slopes of the fits are the Weibull moduli that describe variations in the test results. The effects of porosity and raster orientation on the variations in *σ*_*f*_ and TS were statistically the same for all seven sets of data, i.e., *σ*_*f*_ and TS showed similar dependance on *P* and raster orientation (Fig. 6a,b, Table 1). For *σ*_*f*_ and TS, we observed significantly higher Weibull moduli for high porosity specimens *m* = 36 at *P* = 41 vol.% and *m* = 39 at *P* = 53 vol.%. Whereas, the *m* values were between 18 and 25 for *P* less than 34 vol.%, except for the 0° raster orientation. We observed a decreasing trend in the ${m}_{{\sigma }_{f}}$ with increasing porosity from 15 to 34 vol.%. However, this decrease in ${m}_{{\sigma }_{f}}$ was not statistically significant, since the confidence intervals overlap for the specimens with P = 15, 25, and 34 vol.%. On the origin of the lower ${m}_{{\sigma }_{f}}$ = 18 for P = 34 vol.% were the upper tail deviations toward higher fracture stresses. For example, if we remove the highest two fracture strengths from the data set for P = 34 vol.%, the ${m}_{{\sigma }_{f}}$ increases to 23, which is nominally the same Weibull modulus for the lower porosity specimens ${m}_{{\sigma }_{f}}$ = 24 for P = 15 vol.% and ${m}_{{\sigma }_{f}}$ = 25 for P = 25 vol.%. The Weibull moduli of dense specimens were as low as technical ceramics, specifically M2 with *P* = 13 vol.% had ${m}_{{\sigma }_{f}}$ = 19 and M1 with *P* = 34 vol.% had ${m}_{{\sigma }_{f}}$ = 18, Fig. 7. These low *m* values mean that there is a large scatter in *σ*_*f*_ and TS; for example, the *σ*_*f*_ of M2 with *P* = 13 vol.% ranged from 25.2 to 19.7 MPa, that is a 22% difference between the highest and lowest fracture strength.

The Weibull moduli of *ε*_*f*_ were lower than the *m* values of strength (Fig. 6, Table 1). The ${m}_{{\varepsilon }_{f}}$ values for dense specimens were 11 for M1 and 9 for M2. The dense M1 specimens had *ε*_*f*_ ranging from 2.7% to 4.1 (Fig. 6c), a 34% difference between the highest and lowest *ε*_*f*_. We also observed a decrease in ${m}_{{\varepsilon }_{f}}$ for an increase in porosity from 25 to 53 vol.%, decreasing infill% from 80 to 20 (Table 1). Similarly, the ${m}_{{\varepsilon }_{TS}}$ for M1 was also decreased with increasing porosity from 15 to 53 vol.%.

The Weibull moduli of composites were higher than porous ceramics (Fig. 7). However, in our previous study, we reported Weibull moduli between 46 and 67 for dense ABS in XY raster orientation^11^, which are higher than composite *m* values. Compared to the FDMed ABS, FDMed composites are at the lower end of ABS reliability (Fig. 7, highlighted in yellow). Consequently, there is significant room for improvement in the mechanical reliability of commercial short-fiber-reinforced composites produced using consumer-level FDM systems.

The mechanical properties of FDMed SCFR-ABS differ due to microstructural variations such as distributions of pore size-shape-position, distributions of fiber position-orientation-length-diameter, and variations in deposition path position that are directly related to the distribution of inter-bead pore size. The fracture behavior of FDMed composites can be affected by fiber/matrix interface properties, stress concentrations due to inter-bead pores between the contours and beads, pore-to-pore stress interactions, and pore-fiber stress interactions. Consequently, FDMed composites contain a distribution of failure initiating defect clusters that then introduce variations in *ε*_*f*_ and *ε*_*TS*_. In this context, the decreasing ${m}_{{\varepsilon }_{f}}$ and ${m}_{{\varepsilon }_{TS}}$ with increasing porosity can be attributed to size effects, so that the high porosity specimens contain a low number of crossing beads and low *P* specimens contain more internal beads (Fig. 1). The beads have distribution of inner-bead pores. Accordingly, if one bead fails at a high inner-bead porosity region, the load cannot be transferred to a neighboring bead, which introduces the possibility of low fracture strain in high *P* specimens. Whereas, in low *P* specimens, any low strain failure in beads does not result in catastrophic fracture because neighboring beads can transfer the load. As a results, the probability of low *ε*_*f*_ in low *P* composites is lower compared to higher *P* ones, i.e., the low *P* systems have a narrower *ε*_*f*_ distribution and thus, a higher Weibull modulus than high *P* specimens.

We performed tensile tests on 0° raster orientation to reveal the effect of raster orientation and eliminate raster-to-contour inter-bead pores. The change in raster from 45°/−45° to 0° orientation nearly doubled all the Weibull moduli for *σ*_*f*_, TS, *ε*_*f*_, and *ε*_*TS*_ (Table 1). Specimens with 0° raster also had ~25% higher strength and 66% higher elastic modulus (Table 1). The tested specimens in 0° orientation were printed in batches of ten specimens. The first two sets had average *σ*_*f*_ of ~30.5 MPa, but the last batch had 37 MPa. This last batch is seen in Fig. 6a,b as the upper tail deviation. We censored the top ten values to estimate the Weibull modulus of *ε*_*f*_ and *ε*_*TS*_, since this would otherwise result in an underestimated *m* value. The increase in *m* value and *σ*_*f*_ with the change in raster orientation from 45°/−45° to 0° demonstrates the effects of fiber and inner-bead pore orientation, as well as the inter-bead pore orientation.

**Figure 8:**
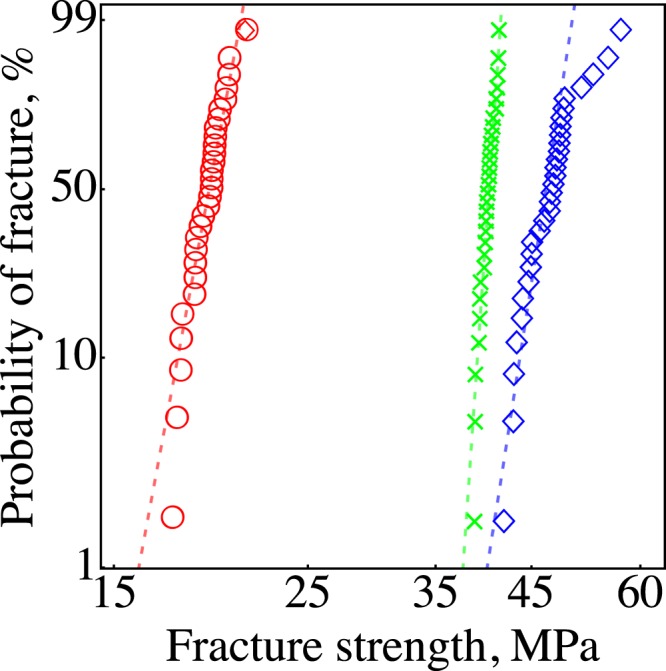
Weibull plot of fracture stress for the extruded filament (◯), M1 filament (×), and M2 filament (◇).

We also performed a large number of tensile tests on the as-received filaments and filaments extruded from the nozzle; these variations in *σ*_*f*_ are shown in the Weibull plot (Fig. 8). These filaments had fibers aligned along their axis that were similar to the specimens with 0° raster orientation. The representative microstructures can be seen for the as-received filament in Fig. 2d and for the extruded filament in Fig. 2e. The Weibull modulus for *σ*_*f*_ of the M1 filament was 62 (49–78), for M2 was 27 (21–36), and for the extruded filament was 23 (18–28); 90% confidence intervals are given in parenthesis. These results show that the FDMed composites with 0° raster can have Weibull modulus as high as the as-received filaments. However, manufacturing the composites in the 45°/−45° raster orientation decreased the *m* value from 62 to 24 for M1 and from 27 to 19 for M2; this discrepancy is due to the change in the structure during extrusion and the defects introduced during FDM. On the other hand, the extruded M1 filament had an ${m}_{{\sigma }_{f}}$ value of 23 (18–28), which is statistically the same as the dense specimens with 45°/−45° raster. The decrease in Weibull modulus and *σ*_*f*_ after extrusion indicates the strong effects of structural changes during extrusion, such as increasing porosity and decreasing fiber length (Fig. 8).

We compared our Weibull fits with fits to normal distribution using Anderson-Darling and Pearson *χ*^2^ goodness-of-fit-tests. The P-values did not indicate a preference for either of the distributions. In general, a good fit to Weibull also showed a good fit to normal distribution. Larger sample sizes are needed to observe a statistically significant difference between Weibull and normal fits. Nonetheless, the mechanical properties of FDMed composites did show deviations from the fitted Weibull distribution, Fig. 6. The origins of these deviations, however, are challenging to explain due to the complex stress interactions between different-sized pores^44^, fibers, and lattice structures.

The exact failure mechanism(s) behind the stochastic fracture in FDMed SCFR-ABS is unclear; however, we believe that the variations in the size of the inter-bead and inner-bead pores caused the scatter in the mechanical properties. Inter-bead porosity depends on the extruded bead thickness and the precision of the deposition path. Fluctuations in a filament’s thickness, fiber content, and porosity cause a variation in the thickness of beads. For example, a relatively porous region of the filament results in thinner beads due to the decreased amount of material at the extrusion tip; this, in turn, increases the inter-bead porosity. In addition, the precision with which beads were extruded along the deposition path was limited by the FDM’s belt system and servo motors. Therefore, beads can be deposited closer to or further apart from each other. If the beads are printed further apart, the size of the inter-bead porosity will be larger. Finally, the inner-bead porosity also fluctuates and causes variations in the fracture strength of the extruded filament (Fig. 8).

It should be noted that we are not reporting the sole effect of internal structure on the mechanical behavior of FDMed composites as the composites were composed of four dense layers (two at the top/bottom), single contour in each layer, and the rectilinear infill structure. That is, at least a third of the specimens were dense for all the composites and the load in low infill samples was mainly carried by the contours and dense top/bottom layers.

A difference in mechanical behavior between the M1 and M2 was also observed. These M1 and M2 were sold as the same filament. The M2 was ~17% stronger than the M1, but the M2 was ~22% less ductile than the M1. These observations show that the process of fiber mixing into ABS and the process of filament extrusion can change the properties of the filaments and thus, the properties of the FDMed materials. Accordingly, homogeneous filaments without any pores that keep their uniformity during extrusion are needed to achieve high mechanical reliability in FDMed materials.

### Simulated stochastic fracture of FDMed SCFR-ABS

We observed a statistically significant increase in Weibull modulus of *σ*_*f*_ for specimens with porosity ≥41 vol.% (Table 1). The origin of this increase in mechanical reliability is difficult to explain experimentally due to the complex structure of the FDMed composites (Fig. 2). However, inter-bead pores are the largest defects and inner-bead pores are the second largest defects in the specimens with infill <100%; thus, the porous infill pattern and bead strength are the main factors that determine the stochastic fracture. In this context, we used a micromechanics-based model to understand the effect of infill density on the stochastic fracture of two-dimensional rectilinear infill patterns. This model estimates the strength distribution for a representative volume element (RVE, Fig. 1c) under uniaxial tensile loading. The strength distribution of the RVE was then used to estimate the *σ*_*f*_ of the larger structure, representative of the experiments (Fig. 1d). Our simulation approach does not explicitly include the microstructural features (inner-bead pores or fibers) observed in real microstructures (Fig. 1), but the effects of these bead-level defects were included indirectly by assuming the fracture strength of the beads was normally distributed. We obtained this *σ*_*f*_ distribution of the beads from the tensile tests on individual beads (Fig. 8, red circles). The mean value and standard deviation of the normal distribution of limit stress are 19 MPa and 0.8 MPa, respectively. As the failure mechanism of the FDMed composites is not known, a structural part of the RVE was assumed to fail when von Mises measure of the average stress value exceeded the limit estimated from Fig. 8. Failure of one structural part was considered to trigger the failure of the specimen, which is widely named as the weakest link. Overall, the simulations showed the effect of infill% ranged from 20% to 80% on the variations of *σ*_*f*_. The details of the simulation can be found in the methods section.

**Figure 9:**
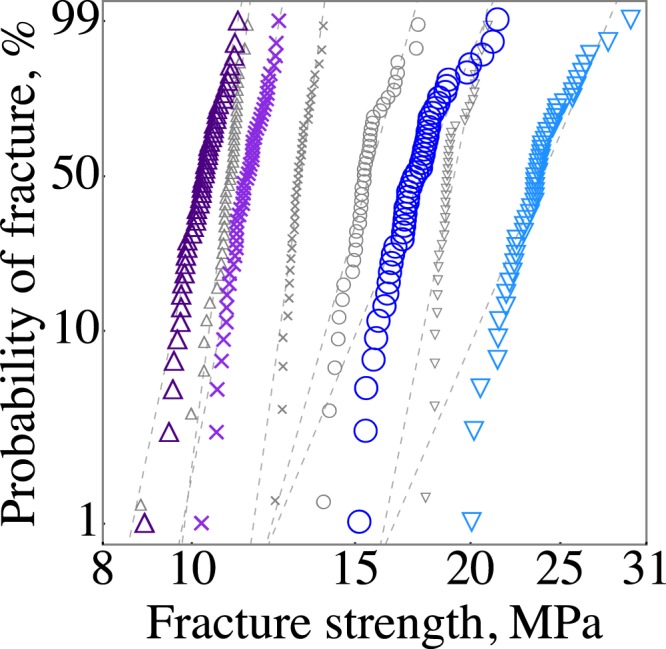
Weibull plot showing the simulated fracture strength (larger and colored markers) and experimental (gray markers) for P (vol.%) = 53 Δ, P = 41 ×, P = 34 ◯, and P = 25 ∇.

The simulations captured the changes in *σ*_*f*_, *m*, and deviations with infill% (Fig. 9). The main failure mechanism of the material was observed to be the breaking of the bond between the crossing beads rather than tension failure of the bead itself. Simulated Weibull modulus was higher ($m\simeq 24$) for infill ≤40% than the modulus ($m\simeq 12$) for infill ≥60%, which is similar to the experimental results (Fig. 9). The simulations also resulted in deviations from the fitted Weibull distribution for 60% and 80% infills. In our simulations, the statistical variation in strength was introduced through material properties, which showed that Weibull modulus is affected by specimen size, failure mechanism, infill%, and the ratio of dense surface layers and low-density infill volume. In the simulations, we omitted geometrical variations related to the bead positions; that is, variations in bead-to-bead distances in experiments were not simulated so that we could mainly investigate the effect of infill%. Extensive simulation studies are needed in the future to clarify the effect of porosity (infill structure) on the stochastic fracture of FDMed SCFR ABS.

A deeper understanding of fracture initiation and propagation in FDMed SCFR-ABS can be achieved in the future by using digital image correlation (DIC) and performing multi-scale cohesive zone model (CZM) simulations. DIC can experimentally reveal the regions in which damage accumulates and fracture occurs. In addition, the internal structure of SCFR-ABS that was obtained by X-ray CT can be used in multi-scale CZM simulations to observe the damage accumulation and failure initiation at micro/meso-scale. These future studies can be used to elucidate the origins of the variations in mechanical properties of FDMed composites.

In conclusion, we performed many tensile tests on FDMed composites and numerical tests on rectilinear lattice structures. The following conclusions were drawn from the experiments and simulations:

1. Five different pores were identified using high-resolution X-ray CT: (a) intentional pores for infill <100%, (b) inter-bead pores, (c) inner-bead pores due to porous as-received filament, (d) inner-bead pores at the ends of carbon fibers, and (e) inner-bead pores due to high-temperature extrusion.
2. Fracture strength of FDMed SCFR-ABS decreased with increasing porosity, which was represented by *σ*_*f*_ = 30 Exp[−0.02 *P*] for 13 vol.% < *P *< 53 vol.%.
3. Experiments and simulations showed that Weibull moduli for *σ*_*f*_ and TS increased for porosity higher than 41 vol.% (infill% ≤ 40). Despite the higher reliability of low infill (40% and 20%) specimens, the fracture/tensile strength of these composites were about half of the strength of the dense composites.
4. Weibull modulus for *ε*_*f*_ and *ε*_*TS*_ decreased with increasing porosity from 25 to 53 vol.%.
5. Change in raster orientation from 45°/−45° to 0° increased Weibull modulus for *σ*_*f*_, TS, *ε*_*f*_, and *ε*_*TS*_, and increased average *σ*_*f*_ by <40%.
6. Extrusion of the 1.75 mm diameter filament through a 0.6 mm nozzle reduced the carbon fiber length by 22% from 117 ± 65 *μ*m to 91 ± 52 *μ*m for one location of the M1 filament and by 48% from 148 ± 76 *μ*m to 77 ± 44 *μ*m at another location.
7. Micromechanics-based simulations showed that the main failure mechanism is the breaking of the beads near the bead-crossing (overlapping beads), which was experimentally observed. Moreover, specimen size, failure mechanism, and infill% affects mechanical reliability of FDMed composites.
8. The following approaches can be used to increase the mechanical reliability of FDMed composites: (a) rectilinear infill pattern can be modified to reduce stress concentration near the 90 °Crossing beads, (b) the size distribution of the inter-bead porosity can be narrowed by using higher precision/accuracy FDM systems, (c) a pore-free filament with uniformly distributed fibers can be used, and (d) longer fibers with a stronger matrix adhesion can be used.

## Methods

### Materials and processing

Two sets of SCFR-ABS filament (1.75 mm diameter) were purchased from 3DXTECH, MI, U.S. We called these sets Material 1 (M1) and Material 2 (M2.). M1 was purchased one year before the M2. Tensile test specimens were produced using an STL file of a rectangular bar 165 × 13 × 3.6 mm. The open source Slic3r program was used to generate the G-code that defined the deposition path. Ten specimens per batch were printed in a RepRap style Prusa FDM system with a 0.6 mm nozzle. We used the following printing parameters: a rectilinear raster orientation of 45°/−45° or 0°, bed temperature of 125 °C, nozzle temperature of 235 °C, an initial layer height of 0.3 mm, and layer height of 0.35 mm. Five different infill percentages (20, 40, 60, 80, and 100%) were used to change the porosity, Fig. 1a,b. The top- and bottom-two layers were set to solid infill (100%) for all the specimens. These top- and bottom-two layers were printed at a rectilinear raster orientation of 45°/−45°, except for the 0° raster specimens that had all the layers in 0° raster. We used a single contour for all the specimens.

### Testing and analysis

We performed tensile tests using an Instron 4204 at a crosshead speed of 1 mm/min for all rectangular bars and filaments. Specimens that broke at the grips were not included in the reported data (about 15% of the tests). Density of each specimen was calculated using the dimensions and weight. A theoretical density of 1.08 g/cm^3^ for composites was calculated using 6.5 vol.% carbon fiber (obtained from X-ray CT analysis, 1.6 g/cm^3^) in ABS (1.04 g/cm^3^). It should be noted that the as-received filament densities can be different at different parts of the filament. We measured the density (using weight and volume) of the M1 filament as 1.23 ± 0.06 g/cm^3^ for a sample size of 15 with one low density of 1.07 g/cm^3^. Density of the M2 filament was 1.28 ± 0.07 g/cm^3^ for a sample size of 10; the manufacturer reported a density of 1.11 g/cm^3^ for M2. We used the theoretical density to be consistent for all the specimens, which should be seen as the lower limit for the porosity calculations. An FEI Quanta 200 scanning electron microscope (SEM) was used to investigate fracture surfaces.

We used an Instron 2710 side action grips with an Instron 2702 serrated jaws to grip and tensile test the as-received M1/M2 filaments and extruded M1 filaments. Thirty tests were performed for each filament, a total of 90 tests. The filaments that broke at or near the grips were invalid and were not included in the Weibull analysis.

We used the maximum likelihood method to estimate the Weibull parameters. Pearson *χ*^2^ and Anderson-Darling (AD) goodness of fit tests were used to compare the fits to Weibull and Normal distributions. The difference between the AD test and Pearson *χ*^2^ test is that the AD test puts more emphasis on the upper and lower tail of the distribution than the *χ*^2^ test.

Segments of 2 cm M1 filament was taken before and after extrusion, which were left to dissolve in a 10 mL reagent grade acetone until fully dissolved. Carbon fibers were allowed to settle on the bottom of a vile and were pipetted onto a glass slide. An Olympus SZX10 Stereo Microscope was used to take images of the fibers at 20x magnification. ImageJ2 software^45^ was used to digitally measure length of ~200 carbon fibers for each histogram in Fig. 5.

### X-ray computed tomography analysis

A ZEISS Xradia 410 Versa X-ray microscope was used for the 3D data acquisition^46,47^. A series of 2D X-ray radiographs were automatically collected across uniformly-spaced angular positions through a 360° rotation, after which each radiograph series was computationally reconstructed using ZEISS XMReconstructor software^48^. Approximately 1600 projection radiographs with a dwell time of 10–15 seconds per view were taken, a total acquisition and reconstruction time of 5–7 hours for each sample in Fig. 2. We were able to identify three-phases in the FDMed composites: polymer matrix, carbon fiber, and pores.

### Simulation procedure

Failure was simulated by a micromechanical model on a representative volume element (RVE) with infills: 20%, 40%, 60%, and 80% (Fig. 1c,d). In the computational homogenization method, the failure statistics in a RVE were used to deduce the failure statistics of a larger domain built out of RVEs. The underlying assumption was that local failure of an RVE triggers the failure of the specimen^49,50^. Constant uniaxial tensile stress *σ* was applied to mimic the conditions of the experiment. The material was modeled to be composed of beads crossing in right angles^51^. Infill ratio defined the size of the RVE. Relative scale *α* = *V*/V^*^ defined the number of RVEs required to fill the specimen. The *α* was set to 60, 140, 220, and 300 representing the experimental infills 20, 40, 60, and 80%, respectively. We estimated the stochastic fracture of the RVE using 50 specimens. Thereafter, the cumulative distribution for the specimen was calculated from cdf (*σ*, *V*) = 1 − [1 − cdf (*σ*, V^*^)]^*α*^.

The simulated fracture strength was calculated by adding 60% of the simulated infill strength to the 40% nominally dense (M1 at P = 15 vol.%) composite strength that was generated from the experimental cumulative distribution function of the dense composite strength. The material was taken to be linearly elastic with Young’s modulus E and Poisson’s ratio *ν* = 1/3. The E for the area of the bead intersection was on average twice the E for the beads, i.e., the darker grey area in Fig. 1c has higher Young’s modulus than the lighter grey area. Young’s modulus was assumed as a lognormally distributed statistical quantity of the mean value <E> = E^*^ and standard deviation = E^*^/5 on scale of the structural part.
